# *Discover mental health*: a journey of growth, achievement, and future aspirations

**DOI:** 10.1007/s44192-024-00081-6

**Published:** 2024-09-09

**Authors:** Julio Licinio, Ma-Li Wong

**Affiliations:** SUNY Upstate Medical University, Syracuse, NY United States 13210

## Introduction

Founded in 2021, *Discover Mental Health* has been an incredible ride! Within just three years, *Discover Mental Health* has evolved from an infant publication into a mature global brand facilitating groundbreaking mental health research, and part of the Discover series, a new family of journals from Springer Nature. This editorial is a look back at the accomplishments of the journal, an acknowledgment of the extraordinary real fruits of labor from our team and our authors, and a preview of the bright future that awaits.

### Birth & growth of *discover mental health*

The vision for *Discover Mental Health* has been to establish a unique innovative hub of research excellence focused on one hand on developing high quality innovative research to further our knowledge of the aetiology of mental health; and secondly on research addressing the development of new interventions. Since its launch, the journal has aimed to publish the most exciting research on mental health, across the full range of biology to intervention. The success of *Discover Mental Health* is a testament to the resolute dedication and talent of our Editorial Board, Section Editors, Authors, and the entire team at Discover. We are sincerely thankful to those who generously gave their time and expertise throughout the years, helping to guide its direction and establish the journal as a diverse and reliable global resource for health professionals and policymakers.

### Milestones and achievements: progress under the leadership of Dr. Julio Licinio

*Discover Mental Health* accomplished significant progress under Dr. Julio Licinio. A key accomplished milestone has been the indexation of the journal in all the major databases including DOAJ, SCOPUS, and PubMedCentral. This is testament to the strength and impact of the research published in *Discover Mental Health* and has significantly raised the profile and the reach of the journal, attracting submissions from leading researchers globally.

A critical contributor to *Discover Mental Health*'s success has been the exceptional efforts of our Section Editors. We extend special thanks to:

- Thang M. Le, PhD, from the Department of Psychiatry at Yale University
- Douglas Teixeira Leffa, MD, PhD, from the Federal University of Rio Grande do Sul and Hospital de Clínicas de Porto Alegre
- Naveen Nagarajan, PhD, from the Department of Human Genetics at the University of Utah
- Giselli Scaini, PharmD, PhD, from The University of Texas Health Science Center at Houston
- Andrea Laurato Sertié, PhD, from Hospital Israelita Albert Einstein in São Paulo
- Krishna C Vadodaria, PhD, from Engrail Therapeutics
- Sudhirkumar Yanpallewar, MD, from the Scientific Review Branch at the National Institute on Drug Abuse

Their experience, expertise, consistency, and dedication to maintaining the journal’s highest standards have been fundamental to *Discover Mental Health* being a successful journal in mental health research. Finally, we are grateful to our Publisher, Jing Wang and to Samuel Winthrop, Executive Publisher, at Discover. Their help, advice, and dedication have been crucial in overcoming the trials of launching and running our journal, and their work has been indispensable to the smooth running of *Discover Mental Health*.

### New chapter: introducing Dr Ma-Li Wong

While we celebrate the successes of *Discover Mental Health,* we are also excited to welcome Dr. Ma-Li Wong as the new Editor-in-Chief. Dr. Wong is experienced and brings fresh perspectives to the journal, and with this, we believe that *Discover Mental Health* will continue to surge during her tenure as Editor-in-Chief. Dr. Wong has big plans and an even clearer vision of the future of *Discover Mental Health*. She hopes to broaden the journal's reach to encompass current and interdisciplinary areas of research in mental health, such as digital mental health, precision psychiatry, and the mental health interfaces with other domains, like neurology, immunology, and public health. Being aware of these advancements allows *Discover Mental Health* to encourage cross-disciplinary collaborations and expedite the translation of research discoveries into clinical strategies benefiting global populations.

### Reach, engagement, and accessibility

While *Discover Mental Health* embarks on this next chapter, it continues its mission to make the research published in the journal more accessible and more impactful through our full Open Access model through which all published material is accessible globally to researchers, clinicians, and the public. This dedication to an open access model is critical for the publication and dissemination of research findings, to enable its use in practice. We also hope in time to improve the journal’s online interaction with our readers in addition to support for open access. Constructed in the best possible way, it is hoped that our website will create an energetic and engaging community around *Discover Mental Health*—encouraging active participation, collaboration, and knowledge sharing between mental health researchers, clinicians, and other stakeholders, all through social and digital media.

### Our gratitude and looking ahead

As we reminisce on the *Discover Mental Health* side of the road, we are truly thankful to everyone we have interacted with, who gave their time to support the success of the journal. Lastly, we thank our authors for allowing us to publish and promote their work, and their ongoing support for the mental health community. It has been your contributions that have kept the journal alive, and we are privileged to be able to continue to publish your work. Especially to our Editorial Board Members and our Section Editors, thank you for your time and guidance. Your commitment to ensuring the journal maintains the highest standards has helped position *Discover Mental Health* as a well-respected and trusted place for research on mental health. Finally, we want to extend our sincere thanks to our readers for your continuing interest and support of the journal. We so appreciate your support and feedback in forming what *Discover Mental Health* has become, and we look forward to continuing to bring you the most current and impactful research in the field. We are optimistic and excited for the future ahead. The world of mental health research continues to expand and *Discover Mental Health* remains uniquely placed to be a part of this ongoing journey. With Dr. Ma-Li Wong at the helm, and continued engagement from our team, authors, and readers, we are confident that the journal will continue to grow and further its mission to identify, disseminate, and help treat mental health issues.

## Conclusion

As we celebrate the journal's achievements and anticipate the future, we are determined to continue to fulfill our commitment to serve as a reliable and forward-thinking outlet for cutting-edge research that will continually inform and influence mental health care in the years to come. We are calling you all to participate in this exciting journey with us as authors, readers, reviewers, and supporting this exciting journal. By working together, we can strive to create a future where mental health is accepted as a basic component of overall life quality, and its evidence-based treatments are provided to everyone in need. Thank you so much for being part of the *Discover Mental Health* community, we are thrilled to share this exciting growth journey with you!

## Data Availability

Not applicable.

